# Analysis of risk factors for catheter-related bloodstream infection in a parenteral nutrition population

**DOI:** 10.1186/cc11988

**Published:** 2013-03-19

**Authors:** I Conrick-Martin, M McGovern, K Boner, J Bourke, E Fitzgerald, R Hone, M Lynch, D Phelan, C Walshe

**Affiliations:** 1 Mater Misericordiae University Hospital, Dublin, Ireland; 2 Harvard School of Public Health, Cambridge, MA, USA; 3 Beaumont Hospital, Dublin, Ireland

## Introduction

Catheter-related bloodstream infection (CRBSI) is a complication of central venous catheters (CVCs) with an attributable morbidity, mortality and cost [1]. We examined patient risk factors for CRBSI in an adult parenteral nutrition (PN) population.

## Methods

The study was carried out in a 525-bed tertiary-referral teaching hospital over a 14-year study period (1997 to 2010). All in-patients referred for PN via CVCs were included. Prospectively collected data were recorded in a specific PN record. The CRBSI audit group met quarterly to review all sepsis episodes, assigning a diagnostic category (CRBSI or non-CRBSI). Patient risk factors for development of CRBSI were examined using a logistic regression model to take account of the dichotomous nature of the outcome. Odds ratios from a model incorporating demographic and clinical data were tested for statistical significance.

## Results

The study population was 1,961 patients in whom 3,213 CVCs were utilised over 19,511 CVC days. There were 256 CRBSI episodes in 216 patients. Median (IQR) patient age was 62 (23), and 58% were male. The incidence of CRBSI decreased significantly (*P <*0.001) during the study period from 16% of patients in the period 1997 to 2003 to 7% in 2004 to 2010. The corresponding rate of CRBSI infection (per 1,000 CVC days) decreased from 18 to 10. There was a significant decrease (*P <*0.001) in numbers of CVCs inserted per patient (from 1.87 to 1.49). Each extra CVC PN day was associated with an increased risk of developing CRBSI of 3.4% (OR 1.034, *P <*0.05). The number of PN CVCs was associated with developing CRBSI (OR 1.218, *P <*0.10). Patient factors significantly associated with CRBSI included perioperative PN use (compared with medical patients) (OR 2.414, *P <*0.01), and male sex (OR 1.952, *P <*0.01).

## Conclusion

This prospective study demonstrated that perioperative PN use was associated with increased risk of CRBSI. The association between CRBSI and CVC PN days is consistent with the theory suggesting benefit to limiting CVC duration and changing from PN to enteral nutrition as soon as appropriate.
